# Atypical Endometriosis: A Comprehensive Systematic Review of Pathological Patterns and Diagnostic Challenges

**DOI:** 10.3390/biomedicines12061209

**Published:** 2024-05-29

**Authors:** Vito Andrea Capozzi, Elisa Scarpelli, Sara dell’Omo, Martino Rolla, Alessandra Pezzani, Giovanni Morganelli, Michela Gaiano, Tullio Ghi, Roberto Berretta

**Affiliations:** Department of Obstetrics and Gynecology, University Hospital of Parma, 43125 Parma, Italy

**Keywords:** endometriosis, atypical endometriosis, endometriosis-associated ovarian cancer, ultrasound and endometriosis, endometriosis and risk of malignancy

## Abstract

Endometriosis is a benign condition affecting women of reproductive age. A potential association with ovarian cancer has been documented. Atypical endometriosis (AE) is characterized by deviations from the typical microscopic appearance of endometriosis, including cytologic and architectural atypia. AE has been recognized as a potential precursor to endometriosis-associated ovarian cancers (EAOC), particularly endometrioid and clear cell subtypes. AE presents challenges in diagnosis due to its diverse clinical and pathological features, often requiring careful histological evaluation for accurate identification. Architectural AE, defined by localized proliferation of crowded glands with atypical epithelium resembling endometrial neoplasia, and cytologic AE, characterized by nuclear atypia within the epithelial lining of endometriotic cysts, are key subtypes. Immunohistochemical and molecular studies have revealed aberrant expression of markers such as Ki67, COX-2, BAF250a, p53, estrogen receptor, progesterone receptor, and IMP-3. Long-term follow-up studies suggest relatively low recurrence and malignant transformation rates among patients with AE, but uncertainties persist regarding its exact malignancy potential and optimal management strategies. Integration of artificial intelligence and shared molecular aberrations between AE and EAOC may enhance diagnostic accuracy. Continuous interdisciplinary collaboration and ongoing research efforts are crucial for a deeper understanding of the relationship between endometriosis and carcinogenesis, ultimately improving patient care and surveillance.

## 1. Introduction

Endometriosis is an estrogen-dependent condition affecting approximately 5–10% of women of reproductive age [1]. Pathophysiologically, endometriosis is characterized by the presence of endometrial glands and stroma outside the uterine cavity, and symptoms such as chronic pelvic pain, dysmenorrhea, dyspareunia, or infertility. Clinical presentation can be highly nonspecific, which often results in misinterpretation and diagnostic delay [2]. Indeed, despite advances in imaging techniques, most recent guidelines still recommend surgical exploration and histological confirmation of endometriotic tissue for definitive diagnosis [3].

Despite its benign nature, endometriosis shares characteristics with malignant diseases, particularly its potential to invade adjacent or distant tissues. Moreover, as early as 1920 and 1953, Sampson and Scott suggested a possible association between endometriosis and ovarian cancer [4,5]. This association poses additional challenges to the diagnostic framework, especially considering the possibility of extra-ovarian disease and its potential for malignancy.

It is estimated that cancer complicates about 0.5–1% of endometriosis cases, with an overall lifetime risk of 1.9% [6,7,8]. The processes underlying the development of ovarian carcinoma from benign endometriosis have gained interest in recent years. In the last decades, extensive research has focused on the potential role of the endometriotic milieu in the carcinogenic process and the identification of malignant precursors. Interestingly, some authors reported that malignant transformation may be associated with the presence of cytological and architectural atypia, defined as “atypical endometriosis” (AE) [8], which may arise in endometriotic tissue under the stimulation of chronic inflammation processes [9]. AE serves as a crucial intermediary entity bridging the spectrum between typical endometriosis and endometriosis-associated ovarian cancers (EAOC), particularly the endometrioid and clear cell subtypes.

Referring to imaging patterns, endometriosis can present in complex scenarios due to the extent of the disease and characteristics of the lesions that may be defined as “atypical”, often leading to challenges in differential diagnosis with malignant tumors. Transvaginal ultrasound plays a pivotal role in diagnosis and management decision-making, typically revealing endometriomas as well-defined, cystic masses with homogeneous low-level internal echoes, often described as a “ground glass” appearance. In general, ultrasound sensitivity and specificity are reported to be as high as 96% and 93%, respectively [3]. However, endometriomas may also exhibit atypical features, including internal echoes or septations, which can resemble features of EAOC. This similarity complicates counseling for patients regarding treatment and follow-up, especially for young women seeking to preserve fertility. Magnetic resonance imaging serves as a second-line tool; however, it may encounter challenges in distinguishing between typical endometriosis and malignant potential [10,11].

This systematic review aims to summarize the current understanding of the atypical aspects of endometriosis, both pathologically and in imaging patterns. In addition, AE prevalence, molecular aspects, and its significance in EAOC pathogenesis are examined. Our goal is to update clinicians on current knowledge and guide them through the complexities of this clinical entity.

## 2. Materials and Methods

The review adhered to the PRISMA (Preferred Reporting Items for Systematic Reviews and Meta-Analyses) guidelines and was registered in the PROSPERO database with registration number CRD42024524405. Three authors (SDO, VAC, and AP) independently conducted a blinded literature search from December 2023 to March 2024, on PubMed, MedLine, Scopus, and Embase. The keywords included “atypical endometriosis”, “endometriosis-associated ovarian cancer”, and “endometriosis with atypical features”. Articles published between 2000 and 2023 were screened. Randomized controlled trials, case series, retrospective, and prospective studies written in English were included. Data on the prevalence of AE, pathological and clinical aspects, association with ovarian cancer, and follow-up were extracted.

The authors independently screened all abstracts and then evaluated the full-length text of eligible articles to extract relevant data. Two additional authors (E.S. and G.M.) discussed and mediated any discrepancies to reach a consensus. All references were analyzed to evaluate additional eligible studies. The researchers reached an agreement about potential relevance by consensus and according to PRISMA statement guidelines [12]. Studies not aligning with the purpose of the study, case reports, redundant studies, abstracts, and articles not in English were excluded.

## 3. Results

### 3.1. Selection Process

**Figure 1:**
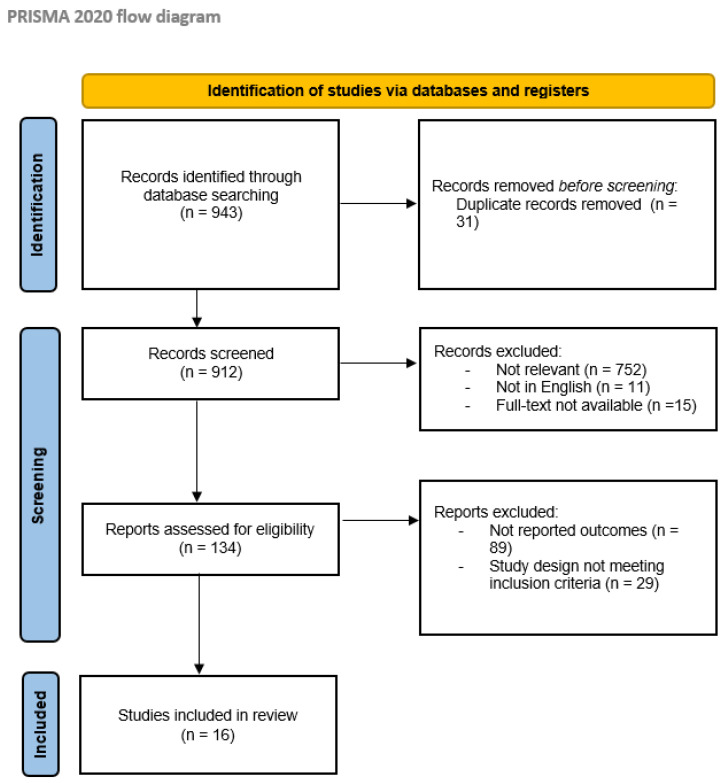
PRISMA flow diagram [12]; http://www.prisma-statement.org/ (27 March 2024).

First, a total of 943 articles published between 2000 and 2023 were identified from the primary database search. Then, 778 articles were excluded due to inconsistencies with the review scope during the screening phase. Finally, 134 articles were considered eligible for the systematic review; of these, 118 were also excluded for the absence of reported outcomes related to AE or the design of the study not in line with the inclusion criteria. Overall, 16 studies were finally selected for this review, 14 retrospective studies [13,14,15,16,17,18,19,20,21,22,23,24,25,26], and 2 prospective studies [27,28]. The selection process is summarized in the PRISMA flowchart in Figure 1.

For better clarity, results are presented in paragraphs. Further details about the included studies are presented in Table 1, Table 2, Table 3 and Table 4.

### 3.2. Prevalence and Histopathology

A total of 6 articles evaluated the prevalence and the histopathology of atypical endometriosis (Table 1). The general prevalence of AE in patients with endometriosis was found to range from <1% [13] to 5.8% [17], while in the case of EAOC, the prevalence was significantly higher, ranging from 22.8 to 34.6% [15,27]. For what concerns the histologic definition of AE, some authors discerned between cellular atypia and architectural atypia. While endometriosis with cellular atypia was more frequently seen in patients without concomitant or subsequent neoplasm, ranging from 4.2% to 71.4% [14,27], the histologic findings of architectural atypia were mainly associated with ovarian cancer, being found in up to 88.9% of cases [27]. Moreover, when comparing AE to EAOC, it appears that the mean age for patients with endometriosis but without malignancy was lower, going from 34 years [16,17] up to 46–49 years [13,15].

**Table 1:** Atypical endometriosis: prevalence and histopathology.

Authors, Year	Design of the Study	Population	Main Outcomes
Wepy et al., 2023 [13]	Retrospective study	4598 patients	- The prevalence of AE was <1%. - The mean age for AE was 46 years (range 26–58 years). - The mean lesion size for AE was 6.5 mm (range 0.5–40 mm). - Locations for AE: ovary (66%), fallopian tube (17%), tubo-ovarian site (3.8%), and peritoneum (3.8%). - 25% of patients with AE showed EAOC. - Most cases of AE displayed moderate cytologic atypia (58%), a low nuclear/cytoplasmic ratio (72%), and hobnail nuclei (67%). The glandular crowded architectural pattern was more frequent in patients with AE and EAOC (67% vs. 7% in patients without malignancy; *p* = 0.001).
Ñiguez Sevilla et al., 2019 [27]	Prospective, observational study	266 patients (159 with endometriosis, 81 with ovarian cancer, 26 with EAOC)	- The general prevalence of atypical endometriosis was 8.8% in cancer-free patients and 34.6% in case of EAOC (*p* < 0.001). - 39.13% of the patients with atypical endometriosis presented EAOC. - AE with cellular atypia was found mainly in patients without neoplasm (71.4% vs. 28.6% in patients with EAOC), while architectural atypia was seen more in patients with ovarian cancer (88.9% vs. 11.1% in patients with endometriosis alone) (*p* = 0.009).
Lenz et al., 2021 [14]	Retrospective study	61 patients (40 with DIE, 5 with atypical ovarian endometriosis, 16 controls without endometriosis)	- The overall prevalence of AE was 4.2%. - All cases of AE showed cytologic atypia with inflammatory features.
Ogawa et al., 2000 [15]	Retrospective study	127 patients with primary ovarian carcinoma.	- 37 patients had concomitant endometriosis (29.13%) and were younger than patients without endometriosis (mean age 48.9 years vs. 52.5 years; *p* = 0.105). - 29 patients with endometriosis showed AE (78.4%). Weak cytologic atypia was reported in 22 cases (59.5%), while strong atypia in 7 patients (18.9%).
So, et al., 2021 [16]	Retrospective study	98 patients with AE.	- The general prevalence of AE was 0.8% (98 of 13,074 patients diagnosed with endometriosis). - The mean age for AE was 34.8 ± 7.3 years. - Locations for AE: right ovary (55.1%), left ovary (35.7%), both ovaries (8.2%), pelvic peritoneum (1%). - 12.3% of AE cases were associated with malignant ovarian tumors (3.1% with borderline tumor, 9.2% with invasive carcinoma). - The mean diameter of the ovarian cyst was 7.2 ± 2.7 cm for AE alone, while it was 8.8 cm in the case of AE with ovarian malignancy (*p* = 0.025).
Bayramoğlu et Düzcan, 2001 [17]	Retrospective study	147 patients (137 ovarian endometriosis, 10 EAOC).	- The general prevalence of AE was 5.8%, while reactive atypia was found in 30.8% of cases. - The mean age for AE was 34.1 years; no significant difference was found between typical endometriosis, atypical endometriosis, and reactive atypia (*p* < 0.05). - Hobnail cell metaplasia was present in 28.6 of AE cases, in 20.5% of reactive atypia cases, and only 3.1% of cases without atypia (*p* < 0.05).

AE, atypical endometriosis; EAOC, endometriosis-associated ovarian cancer; DIE, deep infiltrating endometriosis.

### 3.3. Immunohistochemical and Molecular Features

Nine articles focused on the immunohistochemical and molecular features potentially associated with atypical endometriosis. The analysis included markers such as Ki67, p53, COX-2, BAF250a, IMP-3, and hormonal receptors, assessed directly on atypical endometriosis slides from ovarian or extra-ovarian lesions (Table 2). Four articles evaluated the role of the proliferation index Ki67, revealing labeling ranging from <1% to 10% in atypical endometriosis [13,15,19,27].

A single study evaluated the expression of COX-2, reporting a significantly lower expression in AE compared to typical endometriosis (*p* < 0.001); moreover, COX-2 positivity was significantly higher in endometriosis with cellular atypia than with architectural atypia (80% vs. 20%, *p* = 0.089) [27].

**Table 2:** Atypical endometriosis: immunohistochemical and molecular features.

Authors, Year	Design of the Study	Population	Main Outcomes
Ñiguez Sevilla et al., 2019 [27]	Prospective, observational study	266 patients (159 with endometriosis, 81 with ovarian cancer, 26 with EAOC) Sites: not specified	**Ki67** The Ki-67 was 2.63% in patients with typical endometriosis and 14.61% in patients with atypical endometriosis (*p* < 0.001). In AE with cellular atypia was 5.93%, while in AE with architectural atypia was 22.58% (*p* = 0.004). **COX-2** COX-2 was expressed in 96.8% of typical endometriosis and 60% of atypical endometriosis (*p* < 0.001). In AE with cellular atypia, COX-2 was positive in 80% of cases versus 20% of AE with architectural atypia (*p* = 0.089). **BAF250a** The loss of expression of BAF250a was reported in 3% of typical endometriosis vs. 23.8% of atypical endometriosis (*p* = 0.004). In AE with cellular atypia, BAF250a was negative in 9.1% of cases, while in AE with architectural atypia in 40% of cases (*p* = 0.149).
Wepy et al., 2023 [13]	Retrospective study	4598 patients 33 cases of AE tested with immunohistochemistry from different sites (ovaries, tubes, peritoneum)	**Ki67** In atypical endometriosis, the proliferation index was low, with labeling ranging from <1% to 10%, with a median of 5%. **p53** 100% of cases of AE demonstrated wildtype (not mutated) p53. **ER/PR** AE was frequently positive for ER and PR, respectively, 97% and 76% of cases.
Ogawa et al., 2000 [15]	Retrospective study	127 patients with primary ovarian carcinoma. Sites: ovaries	**Ki67** The mean values of the Ki67 index were: 23.1 ± 3.29 in ovarian carcinoma, 2.7 ± 0.90 in typical endometriosis, and 9.9 ± 1.73 in atypical endometriosis. Significant differences were reported between AE and carcinoma (*p* < 0.05), but also between AE and typical endometriosis (*p* < 0.05).
Stamp et al., 2016 [18]	Retrospective study	42 patients (35 cases of EAOC and 8 cases of AE without cancer). Sites: not specified	**BAF250a** The loss of expression of BAF250a was reported in 14 of 35 cases of EAOC (40%) and in this group of patients, 6 of 10 had BAF250a loss also in AE contiguous to the tumor (60%). Diversely, all cases of AE in cancer-free patients showed retention of BAF250a staining (no loss of expression).
Lenz et al., 2021 [14]	Retrospective study	61 patients (40 with DIE, 5 with atypical ovarian endometriosis, and 16 controls without endometriosis). Sites: ovaries, DIE, lymph nodes	**Ki67** In the AE group, the Ki67 labeling ranged between 5 and 50% (on average 32%). **p53** In the AE group, a strong nuclear p53 expression was reported, with glandular cell positivity ranging from 20% to 28% (on average 26%). Compared to the control group, differences reached statistical significance (*p* < 0.001). **ER/PR** In the AE group, the expression of ER was 56% (range 40–70%), while the expression of PR was less than 1%, significantly lower than the control group (*p* < 0.001).
de la Cuesta et al., 2022 [19]	Retrospective study	47 patients (17 EAOC, 6 atypical endometriosis, 17 controls with endometriosis, 7 controls with normal endometrium). Sites: not specified	**Ki67** The mean Ki67 positivity was 7.5 ± 7.0 in atypical endometriosis, 4.2 ± 8.9 in EAOC, 29.2 ± 13.5 in regular endometriosis, and 15.3 ± 16.3 in regular endometrium. Among the groups, the difference was statistically significant (*p* < 0.001). **p53** p53 overexpression was reported in 100% of atypical endometriosis cases and 14 of 17 EAOC (82.4%). The mean values for p53 positivity were 5.7 ± 5.3 for AE, 23.2 ± 31.6 for EAOC, 0.2 ± 0.5 for regular endometriosis, and 00 ± 0.0 for regular endometrium. Among the groups, the difference was statistically significant (*p* < 0.001).
Bayramoğlu et Düzcan, 2001 [17]	Retrospective study	147 patients (137 ovarian endometriosis, 10 EAOC). Sites: ovaries	**p53** In the atypical endometriosis case, no p53 overexpression was found.
Andersen et al., 2018 [20]	Retrospective study	83 patients (19 benign endometriosis, 11 atypical endometriosis, 9 concurrent endometriosis, 21 EAOC). Sites: not specified	**ER/PR** Expression of ER-β increased during the transition from benign endometriosis to EAOC, while PR expression decreased from endometriosis to EAOC.
Vercellini et al., 2013 [21]	Retrospective study	516 patients (874 samples of excised endometriomas). Sites: ovaries	**IMP3** 7 out of 8 (88%) atypical endometriotic cysts showed staining for IMP3, while in the contiguous endometrial benign cells, the staining was absent. Only one atypical endometriotic cyst was negative for IMP3 in both the atypical and the benign endometrial cells of the epithelial lining.

AE, atypical endometriosis; EAOC, endometriosis-associated ovarian cancer; ER, estrogen receptor; PR, progesterone receptor; DIE, deep infiltrating endometriosis.

BAF250a loss of expression has been observed to occur in up to 60% of cases of AE [18], being more frequent in atypical endometriosis compared to typical cases (23.8% vs. 3%; *p* = 0.004) [27]. A significant difference in BAF250a loss of expression has also been registered between architectural and cytologic atypia (40% vs. 9.1%, *p* = 0.149) [27].

Another potential biomarker that has been investigated is the p53 tumor suppressor, which was found to be overexpressed in AE in up to 100% of cases [14,19], while contradictory findings were reported in other studies [13,17].

The role of hormonal receptors for estrogen (ER) and progesterone (PR) was investigated by three research groups. ER expression is mostly maintained in atypical endometriosis, with reported rates ranging from 56% [14] to 97% [13]. Moreover, Andersen et al. showed that the expression of ER-β increases during the transition from benign endometriosis to EAOC [20]. However, the expression of PR in atypical endometriosis is more debated. One study found PR positivity in up to 76% of AE cases [13], while another study reported positivity in less than 1% of cases [14].

Additionally, Vercellini and colleagues evaluated an innovative marker, the oncofetal protein IMP-3, demonstrating that 88% of atypical endometriotic cysts showed staining, while the staining was absent in the contiguous endometrial benign cells [21].

### 3.4. Malignancy Potential and Recurrence Risk

Three articles described the malignancy potential and the recurrence risk related to the presence of AE (Table 3). In the study by Won, during a median follow-up period of 26.0 months, the AE group showed a significantly higher cumulative recurrence rate compared to the group with ovarian endometriomas (*p* = 0.003) [22]. Tanase et al. reported the case of one patient out of nine who developed recurrent typical endometriosis and finally an endometrioid ovarian carcinoma after conservative surgical treatment of an atypical endometrioma [26]. Conversely, no recurrence of atypical ovarian endometriosis was found by other authors [23,26].

**Table 3:** Atypical endometriosis: Malignancy Potential and Recurrence Risk.

Authors, Year	Design of the Study	Population	Follow-Up Period	Main Outcomes
Won et al., 2020 [22]	Retrospective cohort study	2681 patients (2595 typical endometriosis, 86 atypical endometriosis).	Median duration of 26.0 months, range of 6–138 months.	- Cumulative recurrence rates of ovarian endometrioma at 12, 24, 36, and 60 months were 4.4%, 8.3%, 12.0%, and 21.3%, respectively. In the AE group, the cumulative recurrence rate was significantly higher (*p* = 0.003). - Risk factors for recurrent endometrioma were preoperative CA125 > 48.0 U/mL. - (HR = 2.741; *p* < 0.001), multilocular cyst (HR = 1.909; *p* = 0.016) and the presence of AE (HR = 2.666; *p* < 0.001). - No transformation from AE to carcinoma was recorded during the follow-up.
Tanase et al., 2019 [26]	Retrospective study	9 women surgically treated and diagnosed with atypical endometriosis, over a 12-year period	Median 68 months.	One patient developed recurrent endometriosis and finally endometrioid carcinoma (stage 1C, grade 1) 48 months after cystectomy.
Kim and Hong, 2022 [23]	Retrospective study	41 patients histologically diagnosed with ovarian AE: - 7 patients diagnosed with EAOC and AE - 34 with AE only - 26 patients underwent cystectomy	58.27 ± 33.22 months	Recurrence was suspected in the US in 5 patients out of 26 (19.2%). One of these underwent second-line ovarian cystectomy with a result of typical endometriosis.

AE, atypical endometriosis; EAOC, endometriosis-associated ovarian cancer; US, ultrasound.

### 3.5. Imaging Challenges in Differential Diagnosis

Three articles investigated the role of sonography in the evaluation and differential diagnosis between AE and EAOC (Table 4). Subjective assessment by expert operators demonstrated higher sensitivity (89.73% vs. 64.38%) and specificity (97.15% vs. 96.54%) compared to simple rules [28].

Regarding ultrasound features, Huang et al. noted that AE was associated with smaller cyst size (7.81 ± 2.81 cm vs. 12.68 ± 4.60 cm, *p* < 0.01), more bilateral involvement (28.6% vs. 1.8%, *p* < 0.01), smaller solid components (0.93 ± 1.74 cm vs. 4.82 ± 3.53 cm, *p* < 0.01), fewer (<4 lesions) solid components, and lesions that were wider than taller (12.7% vs. 0%, *p* < 0.01), when compared to EAOC [25]. Similarly, Hernàndez et al. found that EAOC was significantly associated with a larger cyst lesion size (*p* = 0.016), with a cut-off size of 6 cm having optimal sensitivity and specificity (AUC = 0.72, *p* < 0.001), a higher presence of papillary projections, septa, and positive echo-Doppler ultrasound (*p* < 0.001) [24].

**Table 4:** Atypical endometriosis: imaging challenges in differential diagnosis.

Authors, Year	Design of the Study	Population	Main Outcomes
Hernàndez et al., 2022 [24]	Retrospective study	76 patients (59 ovarian endometriosis, 17 EAOC)	- The mean size of the lesion was 6.0 cm in OE and 7.6 cm in EAOC (*p =* 0.016). A cut-off size of 6 cm was found to have optimal sensitivity and specificity (AUC = 0.72, *p* < 0.001). - Papillary projections were present in 11.9% OE and 82.4% EAOC (*p* < 0.001). - Septa were present in 3.4 OE and 70.6% EAOC (*p* < 0.001) - Echo-Doppler signal was positive in 6.8% OE and 88.2 EAOC (*p* < 0.001).
Huang et al., 2022 [25]	Retrospective study	120 patients (63 atypical ovarian endometriosis, 57 OCCC).	- The mean diameter of the cyst was 2.81 cm in AE vs. 4.60 cm in OCCC (*p* < 0.01). - The cyst was unilateral in 71.4% of AE vs. in 98.20% of OCCC (*p* < 0.01). - The loss of ground-glass echogenicity was reported in 6.3% of AE vs. 68.4% of OCCC (*p* < 0.01). - The mean size of internal solid components was 1.74 cm in AE vs. 3.53 in OCCC (*p* < 0.01). - Fewer (<4) solid components were present in 98.4% of AE and 84.2% of OCCC. - Solid components were wider than taller in 12.7% of AE vs. in 0.00% of OCCC (*p* < 0.01).
Saeng-Anan et al., 2013 [28]	Prospective observational study	638 patients (146 endometriomas).	- One-third of endometriomas (39 of 146) were mistaken for malignancy, and in these cases, solid lesions and papillary projection were the most common features (38.5% of the missed diagnoses). - In the diagnostic evaluation, simple rules had a sensitivity of 64.38% and a specificity of 96.54%, while the pattern recognition by an expert operator had a sensitivity of 89.73% and a specificity of 97.15%.

OE, ovarian endometriosis; AE, atypical endometriosis; EAOC, endometriosis-associated ovarian cancer; OCCC, ovarian clear cell carcinoma.

## 4. Discussion

### 4.1. Atypical Endometriosis: Pathological Features, Diagnostic Challenges, and Malignancy Risk

From a histopathological perspective, endometriosis is characterized by the presence of two of the following three features: extrauterine endometrial stromal cells and/or endometrial cell glands, and findings consistent with chronic bleeding [29]. More specifically, ovarian endometrioma is defined as an ovarian cyst with a wall lined with endometrial tissue, containing substantial amounts of clotted and unclotted blood products in its lumen [29]. Alterations in the typical microscopic appearance of endometriosis, including cytologic and architectural atypia, can occur, leading to a diagnosis of AE [30,31].

Cellular or cytologic atypia is characterized by the presence of nuclear atypia within the epithelial lining of endometriotic cysts, such as nuclear stratification, hyperchromatism, and pleomorphism. Instead, architectural atypia or hyperplasia is comparable to endometrial hyperplasia (simple or complex atypia, with or without cytologic alterations) [30]. Extensive research has been conducted on the histologic transition between endometriosis, AE, and EAOC [32], with a proven connection between AE and EAOC, supporting its role as a pre-malignant lesion [32,33]. The criteria for cytologic atypia in endometriosis were first introduced by Czernobilsky in 1979 [34] and supported by LaGrenade in 1988 [32], defined as the presence of eosinophilic cytoplasm, large hyperchromatic or pale nuclei with moderate to marked pleomorphism, an increased nuclear to cytoplasmic ratio, cellular crowding, and stratification or tufting. Hobnail cells could also be occasionally present [31]. However, mild nuclear atypia in endometriotic cysts is not unanimously recognized as a premalignant marker, since cytological atypia is also linked to reactive changes, which present in up to 22% of atypical ovarian endometriomas [31].

However, the World Health Organization currently accepts a mixed definition, defining AE as endometriosis with a localized proliferation of crowded glands lined by atypical epithelium resembling endometrial neoplasia, but also as an alteration in endometriotic cyst lining with stratification, disorganization, and cytologic atypia [35].

Although many past studies have considered cytologic and architectural anomalies in endometriosis as a single entity, efforts have been made to distinguish between these two entities, since their clinical significance and prognostic implications may be different [30]. Indeed, architectural atypia seems to replicate the premalignant nature of atypical endometrial hyperplasia with respect to endometrial cancer, also sharing the same risk factors, namely exposition to hyperestrogenism [36,37].

Various authors have investigated the prevalence of AE in endometriosis-associated ovarian lesions and typical endometriomas, and to our knowledge, only two authors considered the definition of architectural AE, distinguishing its significance compared to cytologic atypia.

In a recent large retrospective study reviewing cases of endometriosis over 11 years, it was found that AE comprised <1% of cases, confirming its rarity, mainly located in the ovary (66%), followed by the fallopian tube (17%), tubo-ovarian site (3.8%), and peritoneum (3.8%). The mean age at presentation was 46 years (range 26–58 years), with a mean lesion size of 6.5 mm (range 0.5–40 mm). Most cases showed moderate cytologic atypia (58%) and a low nuclear/cytoplasmic ratio (72%), with various architectural patterns observed. Hobnail nuclei were present in most cases (67%), with neutrophils associated with atypical glands in 44% of cases. Notably, 25% of patients with AE showed synchronous or metachronous tubo-ovarian neoplasia, with architectural atypia being the most significant alteration in patients with synchronous/subsequent neoplasia [13].

These findings align with a prospective study by Ñiguez Sevilla et al., noting a significant difference between AE with architectural abnormalities, mainly seen in patients with ovarian cancer (88.9%), while AE with cellular atypia constituted the majority of cases of AE and was more frequently found in patients without neoplasms (71.4%). This study reported a general prevalence of AE in neoplasm-free patients of 8.8%, significantly rising to 34.6% in patients with endometriosis-associated neoplasms (*p* = 0.001) [27].

Other case series reported AE prevalence in endometriosis tumor-free population and/or EAOC patients but were based only on the cytologic atypia criteria.

Lenz et al. conducted a retrospective study in 2020 on 40 cases of deep infiltrating endometriosis. The diagnosis of AE was based on the criteria reported by LaGrenade and Czernobilsky, finding a frequency of cytologic AE of 4.2% (*n* = 5), over 2 years, with no malignant transformations reported. All the cases reported can be defined as cytologic AE, with inflammatory features [14].

Similarly, criteria reported by Czernobilsky and Morris, LaGrenade, and Silverberg were used by Ogawa et al. to review slides from 127 patients with primary ovarian cancer, demonstrating the prevalence, histopathological characteristics, and proliferation activity of endometriosis and AE associated with it. Overall, 37 patients presented concomitant endometriosis (29.13%), and in 29/37 cases, it was atypical (78.4%) [15].

In their retrospective study, So et al. reported that among 13,074 patients diagnosed with benign endometriosis, AE had a prevalence of 0.8%, localized in various sites, with most cases being benign (87.7%), while 12.3% were associated with malignant ovarian tumors [16].

Baryamoglu and Duzcan compared cases of cytologic AE, atypia due to reactive changes, and typical endometriosis, reporting a prevalence of 5.8% for AE in a population of 130 patients (120 cases with benign endometriomas, ten cases of EAOC). However, the prevalence of AE based on the nature of the ovarian lesion was not reported [17].

In summary, diagnosing AE presents significant challenges due to its diverse pathological features and implications for malignancy risk assessment. Differentiating between cytologic and architectural atypia underscores the complexity of accurately identifying this condition. Current evidence suggests that architectural AE plays a significant role as a bridging lesion towards EAOC. Nonetheless, further research is required to elucidate the relationship between AE and malignancy.

### 4.2. Atypical Endometriosis: Immunohistochemical and Molecular Features

The possibility of a transition from endometriosis to carcinoma, particularly ovarian clear cell carcinoma, and endometrioid ovarian carcinoma, was initially hypothesized by Sampson, who described the presence of endometriotic foci around neoplastic tissue in cases of EAOC. Evidence supporting this continuum comes from various molecular and genetic mechanisms, which are elucidated using diverse immunohistochemical and molecular markers [38].

A potential marker indicative of the pre-malignant potential of AE is the expression of Ki-67. High Ki-67 expression indicates increased mitotic activity and is associated with aggressive tumor behavior and poor prognosis [15,27]. Studies have shown significantly elevated Ki-67 expression in patients with architectural AE compared to those with cytologic AE, confirming the stronger association of architectural AE with ovarian cancer [27]. In addition, the median expression of Ki-67 in atypical endometriotic tissue ranged from <1% to 10% in the report by Wepy [13] and from 5 to 50% in the study by Lenz [14]; significant differences in proliferative activity were also observed between carcinoma and AE, between AE and typical endometriosis, and between carcinoma and typical endometriosis [15].

Chronic inflammation and oxidative stress on endometriotic tissue have been suggested as stimuli for the development of AE [9]. Higher expression of COX-2, an inflammatory marker and potential prognostic marker of ovarian cancer, has been observed in typical endometriosis compared to AE (*p* < 0.001). Interestingly, this difference was also noted between cytologic atypia and architectural atypia [27]. This finding supports the reactive nature of cytologic atypia, while architectural atypia appears to be an initial expression of the malignant potential of AE, on its path towards EAOC.

Moreover, somatic mutations in oncogenes and oncosuppressor genes have been investigated in patients displaying AE associated with EAOC.

The loss of BAF250a expression, indicative of the oncosuppressor ARID1A mutation, is significantly higher in AE compared to typical endometriosis (*p* = 0.004) [27]. Interestingly, this loss was shown to be more pronounced in architectural atypia compared to cytologic atypia (40% vs. 9%), suggesting its role in the malignant transformation process, even if this difference was not statistically significant (*p* = 0.15) [27].

In line with these observations, Stamp et al. also reported that the loss of BAF250a expression in cases of AE is associated with BAF250a-deficient EAOC [18].

Among the investigated markers, p53 immunostaining stands out as a significant indicator of malignant potential due to its role as a tumor suppressor gene. In individuals with AE, Lenz et al. (2020) reported a notable increase in robust p53 expression, further supporting the notion of AE as an intermediate stage in malignant transformation [14]. Additionally, elevated levels of p53 expression have been observed in cases of cancer associated with endometriosis, highlighting its role in malignant progression [19]. However, other studies have reported normal p53 expression in their cases [13,17].

Evaluation of hormone receptor expression (estrogen receptor (ER) and progesterone receptor (PR)) is useful in determining the malignant potential of AE. Lower ER and PR expression levels have been observed in AE compared to typical endometriosis [14]. However, in another study, 97% of cases of AE were ER-positive, and 76% were PR-positive [13]. Changes in hormone receptor signaling, such as increased ERβ expression and decreased PGR expression, have been observed in the transition from endometriosis to EAOC [20].

Furthermore, the oncofetal protein IMP3, epigenetically silenced soon after birth, is re-expressed in a series of human malignancies, including clear-cell ovarian cancer, a histotype strongly associated with endometriosis. In 2013, Vercellini et al. retrospectively analyzed a total of 874 excised cysts to determine whether this oncofetal protein is detectable in endometriomas with or without cytologic/architectural atypia. They found that seven out of eight atypical endometriotic cysts showed staining for IMP3 in the cytoplasm of the atypical endometrial cells, suggesting its potential as a marker of pre-malignant lesions. Immunohistochemical staining for IMP3 could be a reliable test to help discriminate between benign conditions and cytological/structural atypia, aiding in identifying patients at risk of progression to invasive endometriosis-associated carcinoma [21].

Overall, the available evidence suggests that AE may serve as a premalignant lesion predisposing individuals to ovarian cancer, particularly clear cell carcinoma and endometrioid carcinoma. Molecular and genetic mechanisms, along with various immunohistochemical markers, provide insights into the continuum from endometriosis to carcinoma.

### 4.3. Atypical Endometriosis: Malignancy Potential and Recurrence Risk

Despite many studies recognizing AE as a premalignant lesion, the exact malignancy potential of AE compared to typical ovarian endometriosis remains not fully understood, and some concerns may arise about the risk for recurrence and progression to malignancy after AE diagnosis after surgery. Reassuring evidence derives from long-term follow-up case series.

Won and colleagues [22] conducted a retrospective cohort study on 2681 patients who underwent surgery for ovarian endometrioma, including 86 patients with AE, and a median follow-up duration of 26 months. Their report suggests that AE is one of the main risk factors for the recurrence of ovarian endometrioma (HR = 2.666; 95% CI = 1.659–4.284; *p* < 0.001). Other factors for recurrence were higher preoperative CA-125 levels (>48 IU/mL, HR = 2.741; 95% CI = 1.517–4.952; *p* < 0.001) and the presence of more than one locule in the cyst (HR = 1.909; 95% CI = 1.128–3.230; *p* = 0.016). Additionally, women with AE exhibited larger cyst sizes and a higher proportion of multiparity. Although no patients diagnosed with AE developed ovarian malignancy in this study, two typical endometriosis patients experienced borderline malignancy and serous carcinoma, respectively [22].

Similarly, Tanase and colleagues followed up on 9 women who underwent surgical treatment and were diagnosed with AE for a median period of 68 months. Only one patient (11.1%) had a recurrence of endometriosis, which eventually progressed to stage 1C endometrioid carcinoma. There were no cases of recurrence of AE in this study [26].

Regarding the safety of surgical treatment for AE, Kim and Hong evaluated the clinical outcomes after cystectomy in 26 patients with pathological diagnoses of AE after surgery. Recurrence of typical ovarian endometriosis was suspected or surgically confirmed in five patients. Similar to the study conducted by Tanase, no malignant transformation of AE to ovarian cancer was observed during a median follow-up period of 58 months [23].

These findings suggest that while AE is often considered a premalignant lesion, uncertainties persist regarding its precise malignancy potential compared to typical ovarian endometriosis. Concerns regarding recurrence and progression to malignancy post-surgery may arise. However, reassuring evidence from long-term follow-up studies indicates relatively low rates of recurrence and malignant transformation among patients diagnosed with AE, even after conservative surgical treatment.

### 4.4. Atypical Endometriosis: Imaging Challenges in Differential Diagnosis

The typical appearance of an endometrioma is commonly described as a unilocular cyst with homogeneous low-level echogenicity, often referred to as a ground glass pattern. However, these features are only demonstrated in approximately 50–65% of cases [39,40], and the remaining cases may be classified as atypical. In recent years, with increasing attention to non-invasive and minimally invasive treatments, as well as the desire for fertility preservation, conservative management strategies have gained prominence. Consequently, sonography has become a critical diagnostic tool, but it still faces limitations and challenges in assessing the malignancy risk of endometriomas with atypical features. These difficulties are particularly pronounced in older women, as endometriomas in this demographic are more likely to be multilocular, contain solid components (up to 21% in women older than 45 years), and exhibit decreased ground glass echogenicity [41]. Additionally, the fact that CA125 levels are often elevated in endometriosis cases further complicates the malignancy assessment [42].

In 2022, Hernàndez conducted a retrospective analysis investigating diverse risk factors linked to endometriomas for the prediction of EAOC. The study identified several ultrasound features indicative of malignancy, including larger cyst size, presence of papillary projections (>3 mm), increased presence of septa, and positive Doppler signals. Notably, the author highlighted the optimal sensitivity and specificity achieved with a cyst diameter cut-off of 6 cm (AUC = 0.72, *p*-value < 0.001) [24].

Another recent retrospective study by Huang et al. also explored the challenges in distinguishing between endometriomas and ovarian clear cell carcinoma based on sonographic features. The authors underscored the limited role of the IOTA risk score in distinguishing endometriomas from endometriosis-associated ovarian cancer, reporting that age, cyst diameter, loss of ground glass echogenicity, and solid component size (greater than 1.27 cm) are independent risk factors for malignant transformation [25].

Similarly, in 2013, Saeng-Anan et al. retrospectively assessed the accuracy of ultrasound in differentiating endometriomas from ovarian cancer. They noted that one-third of endometrioma cases display atypical patterns that can be mistaken for malignancy, with solid masses and papillary projections being the most common features (38.5%) to mimic ovarian cancer [28].

In conclusion, while ultrasound remains a vital tool in diagnosing endometriomas, its efficacy in assessing the malignancy risk of atypical cases poses significant challenges. The presence of atypical patterns, including solid masses and papillary projections, underscores the importance of integrating clinical and imaging findings for accurate diagnosis and appropriate management decisions.

### 4.5. Implications for Clinical Practice and Future Directions

We acknowledge that the strength of our observations regarding AE occurrence, clinical relevance, and prognostic implications is limited mainly due to the heterogeneous definition of AE across different studies and the predominantly retrospective nature of the sources included. However, some meaningful implications for clinical practice can be derived from the wide synthesis of current evidence reported in our review.

The available evidence supports the role of AE as a precursor to EAOC. The involvement of AE in the continuum between endometriosis and carcinogenesis is evidenced by the higher prevalence of AE in EAOC cases compared to typical endometriosis, as well as molecular characteristics of AE including Ki67 expression, overexpression of p53, loss of function of tumor suppressors such as ARID1A, and innovative biomarkers of potential malignancy such as IMP3. In this context, cytologic atypia may represent an initial and potentially reversible expression of the inflammatory milieu of endometriosis, while architectural AE in particular emerges as analogous to atypical endometrial hyperplasia in relation to the development of endometrial cancer. This hypothesis offers several valuable insights. Shared risk factors and natural history enhance our understanding of the pathogenesis of endometrioid and clear-cell ovarian cancer, potentially influencing clinical practice.

The idea that endometriosis could lead to cancer has a different underlying process compared to the typical development of high-grade serous ovarian cancer. This notion aligns with Kurman’s theory of ovarian malignancies, which divides them into two types: type I and type II. Type II ovarian cancers, which include EAOC, follow a different path compared to the more common type I cancers [43]. Additionally, EAOC and endometriosis share a common origin: the endometrium [44,45].

In line with this hypothesis of a common origin, the most recent ESGO-ESMO-ESP guidelines on ovarian cancer mention extending molecular stratification from The Cancer Genome Atlas for endometrial cancer to endometrioid and clear cell ovarian cancer, although the prognostic significance and therapeutic implications of this classification in ovarian cancer are yet to be fully assessed [46].

The clinical significance of these observations in preventing and managing the risk of ovarian cancer occurrence is limited due to the absence of early diagnosis strategies compared to endometrial abnormalities. However, considering the role of retrograde menstruation in endometriosis and EAOC pathogenesis, as well as the impact of hormonal status on endometrium-derived cells, preventive strategies gain significance [47].

In this context, epidemiological data indicating a protective effect of tubal ligation specifically against endometrioid carcinoma and the protective effect of prolonged oral contraceptive use emerge as potential strategies to counsel patients with endometriosis about prevention and risk reduction concerning EAOC.

Concerning preoperative risk assessment and prediction of EAOC in patients with atypical endometriosis lesions, deviating from the classic pattern recognition of endometriomas, traditional imaging demonstrates limited accuracy in distinguishing between atypical yet benign lesions and EAOC. Integration of artificial intelligence could potentially offer valuable assistance in this context, as also proven in other contexts [48]. Shared molecular aberrations between AE and EAOC may enhance the accuracy in distinguishing between atypical yet benign lesions and EAOC. The literature reports indicate an integrated evaluation facilitated by machine learning and testing through artificial intelligence, demonstrating non-negligible specificity and sensitivity [49].

## 5. Conclusions

In conclusion, endometriosis is a common benign gynecological condition, yet it is associated with rare but significant premalignant and malignant ovarian lesions, which may fall under the broader category of AE. The identification of atypical pathological patterns within endometriosis has become more defined, with specific diagnostic criteria emerging. Recent research has emphasized AE as a potential precursor to malignancy, characterized by abnormal cytology and architecture. Immunohistochemical and molecular studies have uncovered underlying biological mechanisms, providing promising diagnostic markers with prognostic value and potential implications for prevention and early diagnosis strategies. Long-term follow-up studies offer reassurance regarding relatively low recurrence and malignant transformation rates among patients with AE. Nevertheless, uncertainties persist regarding its exact malignancy potential and optimal management strategies. While imaging techniques, especially ultrasound, play a crucial role in diagnosis, challenges persist in distinguishing atypical features from malignancies. Continuous interdisciplinary collaboration and ongoing research efforts are crucial for a deeper understanding of the intricate relationship between endometriosis and carcinogenesis, ultimately enhancing patient care and surveillance.

## Data Availability

The data that support the findings of this study are available on request from the corresponding author (M.R.).
